# Chromatographic analysis of triple cough therapy; bromhexine, guaiafenesin and salbutamol and pharmaceutical impurity: in-silico toxicity profile of drug impurity

**DOI:** 10.1186/s13065-024-01122-5

**Published:** 2024-01-27

**Authors:** Marco M. Z. Sharkawi, Mark T. Safwat, Eglal A. Abdelaleem, Nada S. Abdelwahab

**Affiliations:** 1 Pharmaceutical Analytical Chemistry Department, Faculty of Pharmacy, Beni-Suef University, Alshaheed Shehata Ahmed Hegazy St., Beni-Suef, 62514 Egypt; 2 Pharmaceutical Chemistry Department, Faculty of Pharmacy, Nahda University in Beni-Suef (NUB), Bayad Al Arab, New Beni-Suef City, Beni-Suef, 62764 Egypt

**Keywords:** Bromhexine, Salbutamol, Guaiafenesin, High performance liquid chromatography, Thin layer chromatography, In-Silico study

## Abstract

Bromhexine (BR), guaiafenesin (GUF) and salbutamol (SAL) are formulated as Ventocough syrup® (with and without sugar), labeled to contain propyl paraben and sodium benzoate as inactive ingredients. They are used to make coughing more productive and easier. A crucial element and a major issue in the pharmaceutical industry is the control of organic related impurities to obtain safe and effective treatment. Guaiacol (GUL) is reported to be GUF related impurity that was proved to be extremely toxic (toxic rating class 5), and its use should be banned. In this work, In-Silico study and ADMET estimation were conducted to predict GUL pharmacokinetic properties and its toxicity profile. Additionally, two chromatographic methods were conducted to analyze the studied components along with GUF impurity in the presence of the labeled dosage form excipients. The In-Silico study assured that GUL has oral rat acute toxicity and it is considered to be skin sensitizer. On the other hand, the developed TLC- densitometeric method depended on using a mobile phase mixture of hexane: methylene chloride: triethylamine (5.0:6.0:0.3, by volume) as a developing system. UV-Scanning was performed immediately at 275 nm for SAL, GUF and GUL, while scanning at 310 nm was used for scanning BR. Linearity was established in the ranges of 0.25–4.0, 0.25–4.0, 0.5–8.0 and 0.1–1.6 µg/band for BR, SAL, GUF and GUL, respectively. In the developed HPLC method, separation was performed on X-Bridge® C_18_ column (250 × 4.6 mm, 5 μm) using a solvent mixture of 0.05M disodium hydrogen phosphate pH 3 with aqueous phosphoric acid: methanol (containing 0.3%, v/v triethylamine) (40:60, v/v). Detection was done at 225 nm and separation was achieved within 10 min. Linearity was proved in the range of 2–50 µg/mL for the proposed drugs. Validation of the developed methods was done and all the calculated parameters were within the acceptable limits recommended by ICH guidelines. After that, methods were used to examine the potency of the selected marketed dosage forms and concentrations of all drugs were within the acceptable limits. Additionally, complete separation between the studied drugs and the additives were observed. The developed methods can be used during routine quality control analysis of the proposed drugs when the required issues concern on sensitivity, selectivity and analysis time.

**Graphical Abstract:**

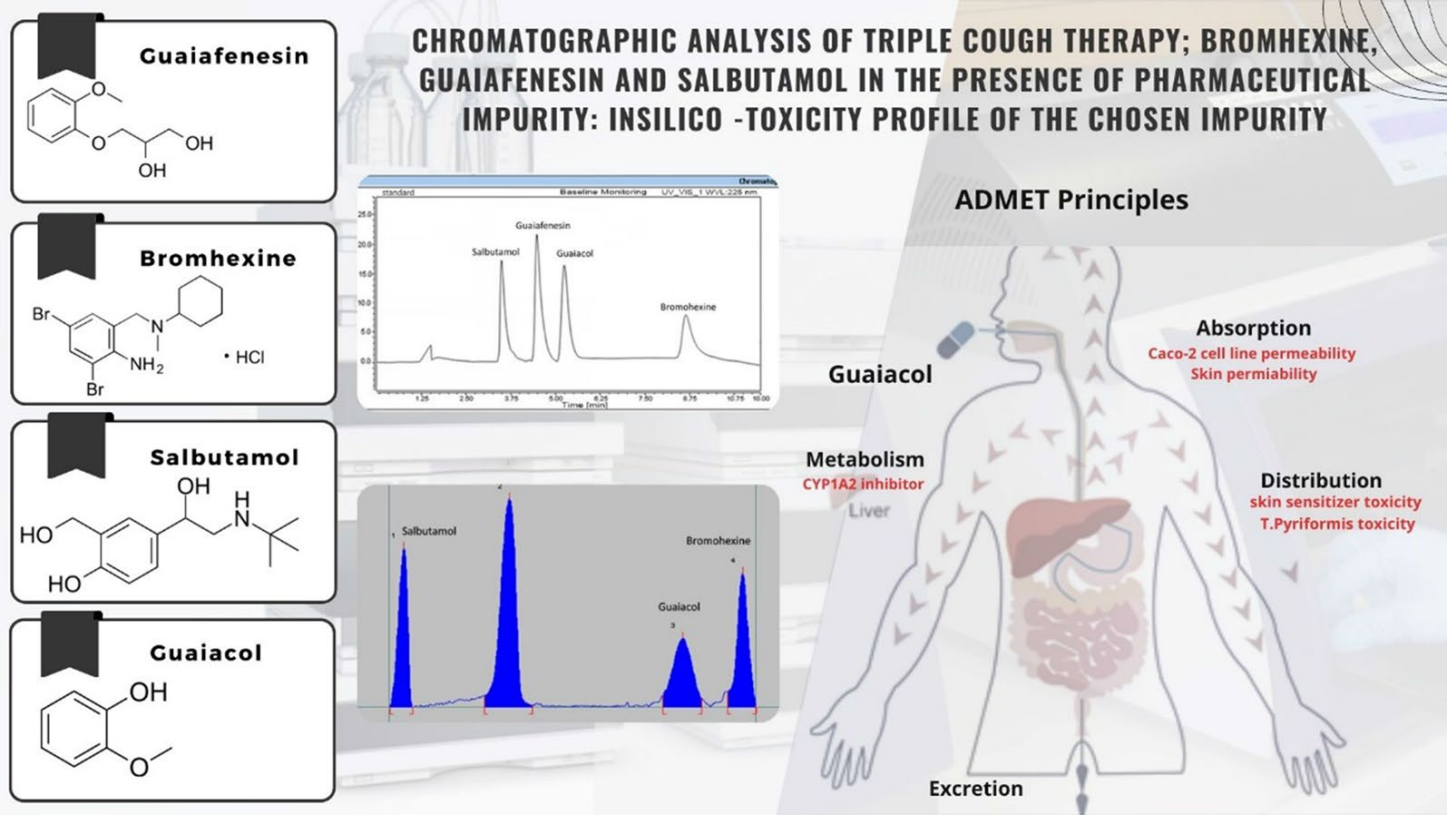

**Supplementary Information:**

The online version contains supplementary material available at 10.1186/s13065-024-01122-5.

## Introduction

Cough is a reflexive response that helps to remove irritants and foreign particles from lower and upper respiratory systems. It is classified according to its type as productive cough or non-productive cough while according to the duration; it is classified into acute, sub-acute or chronic cough. Cough treatments include antitussives, antihistamines, expectorants, proteolytic enzymes, mucolytics, and bronchodilators [1].

Bromhexine (BR) is a mucolytic agent, it increases the body's ability to expel sputum from the respiratory tract by increasing the production of serous fluid in the respiratory tract, making the sputum more thin and less viscous, thus facilitates its excretion [1, 2]. Guaiafenesin (GUF) works as expectorant. It has distinct effects on increasing sputum volume and decreasing its viscosity. Moreover, it facilitates the respiratory tract's sputum flow to the outside, aiding the ciliary action to expel the sputum [1, 3]. Salbutamol (SAL) is short-acting beta agonists (SABA), it acts on the beta-2-receptor, which is common in the bronchial smooth muscles and helps the bronchi to dilate. This is useful in cough accompanied by bronchospasm [1, 4]. Combination of the three drugs is administrated to facilitate productive coughing. They are formulated as ventocough® syrup (with and without sugar) which is labeled to contain both propyl paraben and sodium benzoate as inactive substances. It is worthy noticed that, drug additives need a careful strategy for patient safety because their improper use may induce health risks to the consumers such as allergies, diabetes, obesity, and metabolic diseases [5].

On the other hand, Guaiacol (GUL) is 2-methoxyphenol; in British (BP) [6] and United States (USP) [7] Pharmacopeias it is stated to be GUF-related substance and impurity. Clinical studies [8] have reported that GUL causes many comprehensive health risks including tachycardia, dyspnea, blindness, hyperactivity, myoclonus, abdominal distension, hematuria hepatic and renal necrosis, hemorrhages, pulmonary edema and bladder clotting. It is reported that GUF sample must be discarded when GUL or GUI β-isomer is identified [7].

Referring to the literature review, different analytical methods have been reported for analysis of BR, GUF and SAL individually or in combined dosage forms and in different matrices. BR and GUF were concurrently determined by chemometric [9], HPLC [10] and LC/MS/MS [11] methods while for BR and SAL, they were analyzed together by spectrophotometric [12–15], TLC [16] and HPLC [17] methods. Also, GUF and SAL were determined by chemometric [18], TLC [19] and HPLC [18, 20–24] methods. BR, SAL and GUF were analyzed in different matrices along with other drugs by HPLC [25] and LC/MS/MS [26] methods. There was only one reported work depending upon using three chemometric models for analysis of BR, SAL, GUF and GUL [27]. Till now, there was no reported TLC-densitometeric method for analysis of the three drugs in their available marketed dosage form. Moreover, no chromatographic methods were published for the analysis of the studied drugs along with GUF toxic impurity.

This study aimed to develop simple, sensitive, rapid and accurate TLC-densitometeric and RP-HPLC methods for the simultaneous analysis of BR, SAL, and GUF with the sensitivity and the selectivity required for their analysis in their marketed dosage form without interference from syrup additives. Moreover, the studied drugs were analyzed along with GUF toxic impurity with high sensitivity and selectivity.

Also, and for the first time, In- Silico study and ADMET estimations were conducted for studying of pharmacokinetic parameters and toxicity profile of GUL. The developed TLC-densitometeric approach can be considered superior when economic considerations and sensitivity are the essential requirements while HPLC one is the approach of choice when accuracy and precision are the concerning situations.

## Experimental

### Instruments

#### For TLC-densitometeric method

-The TLC-densitometer (CAMAG, Muttenz, Switzerland) was used with the aid of the winCATS software (Version 3.15; CAMAG) for data manipulation. A Linomat V applicator and a 100.0 µL syringe (CAMAG, Muttenz, Switzerland) were employed to apply the samples. The slit dimensions were 6 × 0.45 mm, with a scanning speed of 20 mm/s, absorbance was the used scanning mode, and a deuterium lamp as the radiation source (CAMAG, Muttenz, Switzerland).

-A UV lamp with a short wavelength of 254 nm (Vilber Lourmat, Marne La Vallee, Cedex, France) was used until the appropriate mobile phase was reached.

-For the stationary phase, TLC aluminium plates (20 × 10 cm) coated with 0.25 mm Silica gel 60 F254 (Merck, Darmstadt, Germany) were used.

#### For RP-HPLC method

Dionex Ultimate 3000 RP-HPLC (Thermo Fisher Scientific, Massachusetts United states) was employed. It came with a Quaternary solvent supply pump, an auto sampler, and a diode array detector. The X-Bridge® C_18_ column (250 × 4.6 mm, 5 µm) ( Waters Corp, Milford, Massachusetts, United States) was used as a stationary phase for development and quantification. The output signal was examined and evaluated using Chromeleon software.

#### Other instruments

- ENTRIS224-1S, Electronic balance (Sartorius AG, Goettingen, Germany).
- Elmasonic S 60 H ultrasonicator (Elma Company, Singen, Germany).
- 250 VM vortex mixer (Hwashin Company, Seoul, Korea),
-A 0.45 µm Millipore membrane filter (Merck Life Science, Ildefonse Vandammestraat 5/7B, 1560 Hoeilaart, BELGIUM) was used to filter the mobile phase.
-Rongtai variable volume micropipette Volume: 1–100.0 µL (Shanghai Rong Tai Biochemical Engineering Company., Mainland, Shanghai, China).

### Samples and reagents

(a) Bromhexine hydrochloride (BR) sample was purchased from Sigma-Aldrich (Merck Life Science BV, Ildefonse Vandammestraat 5/7B, 1560 Hoeilaart, BELGIUM) with a purity of 98.96 and 98.73% according to the results of the developed TLC-densitometric and HPLC methods, respectively.

(b) Guaiafenesin (GUF) sample was supplied from PHARCO Pharmaceuticals (Alexandria-Cairo Desert Rd. Km 31, Amriya, Alexandria, Egypt) and its purity was found to be 98.59% and 99.76% according to the analysis results of the developed TLC-densitometric and HPLC methods, respectively.

(c) Salbutamol (SAL) sample was bought from SEDICO Pharmaceutical (1st Industrial Zone، 6th of October, Giza District, Giza Governorate, Egypt) with labeled purity of 99.76% and 99.09% when it was analyzed by the developed TLC-densitometric and HPLC methods, respectively.

(d) Guaiacol (GUL) (LOBA Chemie, India) with purity of 99.82% and 99.20% according to the suggested TLC-densitometric and HPLC methods, respectively.

(e) Ventocough ® syrup, was labeled to contain the following amounts of the studied drugs for each 5 mL:

- 1.2 mg salbutamol sulphate (eq. to 1mg salbutamol).
- 2 mg bromhexine.
- 50 mg guaiafenesin.

Its batch number was 200819 (for ventocough without sugar) and 220318 (for ventocough with sugar) and they were manufactured by the Egyptian company (Delta Grand Pharma (DGP) for Pharmaceutical industries, 10th of Ramadan City, Ash Sharqiyah, Egypt). They were purchased from the local market.

(f) Chemicals and solvents that used throughout this study were methanol (HPLC grade, Fisher, Southborough, UK, batch number 232687), methylene chloride (Adwic, Egypt, batch number MO58111), hexane (Piochem, Egypt, batch number H13062120005), anhydrous disodium hydrogen phospahate (lanxess, German), orthophosphoric acid (Piochem company for laboratory fine chemicals, Egypt), and triethylamine (Piochem company for laboratory fine chemicals, Egypt).

### Procedure

#### TLC-densitometeric chromatographic conditions

A Camag Linomat V applicator was used to apply samples onto TLC plates (20X 10 cm) as bands of 6.0 mm width. The bands were 5 mm apart and 10 mm from the bottom end of the plate. The chromatographic tank was saturated with a mobile phase mixture of hexane: methylene chloride: triethylamine in the ratio of (5.0:6.0:0.3, by volume) at room temperature for 10 min. After development, the separated bands were scanned at 275 nm for SAL, GUF and GUL while 310 nm was used for BR detection. The slit dimensions were 6 × 0.45 mm.

#### HPLC chromatographic conditions

30 µL of each sample was injected in triplicates to the stationary phase (X-Bridge® C_18_ column) (250 × 4.6 mm, 5 μm). The mobile phase used was 0.05M disodium hydrogen phosphate pH 3 with aqueous phosphoric acid: methanol (containing 0.3% triethylamine) (40:60, v/v). The column temperature was set at 25 ◦C and the flow rate was altered along the separation from 1.5–2 mL/min following an optimized program, Table 1. Scanning was performed at 225nm and the run time was adjusted at 10 min.

**Table 1:** Program of changing mobile phase flow rate for the developed HPLC method

Time (minutes)	Flow rate	MeOH ( containing 0.3% Triethylamine)	% 0.05M disodium hydrogen phosphate solution
0–4.5	2	60	40
4.5–5.5	1.5	60	40
5.5–5.6	2	60	40
5.6–10	2	60	40

#### Solutions

##### Stock standard solutions

Stock solutions of BR, SAL, GUF, and GUL (1000 µg/mL) were separately prepared in 10 mL four calibrated flasks using methanol as a diluent.

##### Working standard solutions

For TLC-densitometeric method, working standard solutions of BR (500 µg/mL), SAL (500 µg/mL) and GUL (200 µg/mL) were prepared separately into three different 10 mL volumetric flasks in methanol from their previously described stock standard solutions.

For RP-HPLC, working standard solutions of BR (100 µg/mL), SAL (100 µg/mL), GUF (100 µg/mL) and GUL (100 µg/mL) were prepared from their corresponding stock standard solutions into four separate 10 mL volumetric flasks and the volume was completed with methanol.

##### Laboratory prepared mixtures

Six different mixtures of the studied components were prepared for each of the developed methods.

For TLC-densitometric method, different aliquots of their prepared standard stock solutions (1000 µg/mL) were transferred to different 10 mL volumetric flasks containing the following concentrations of GUF, BR, SAL and GUL in order: (50, 25, 25, 10 µg/mL), (100, 50, 50, 20 µg/mL), (300, 150, 150, 80 µg/mL), (500,250, 250, 120 µg/mL), (600, 300, 300, 140 µg/mL), and (800, 400, 400, 160 µg/mL) and the volume was completed to the mark with methanol.

For HPLC method, mixtures were prepared by transferring different ratios of GUF, BR, SAL and GUL from their stock (1000 µg/mL) and working (100 µg/mL) solutions to series of 10 mL volumetric flasks containing the following concentrations in order: (10, 2, 3, 2 µg/mL), (4,10, 2, 3 µg/mL), (2, 4, 4, 5 µg/mL) (30, 20, 15, 10 µg/mL), (40,30,20,15 µg/mL), (50,50,30,20 µg/mL) and the volume was adjusted by methanol.

##### Pharmaceutical formulation solutions

For TLC-densitometeric method, two sample solutions each of ventocough® syrup (with sugar) and (without sugar) were prepared separately in methanol by transferring accurately two different volumes (0.1 and 2 mL) of each dosage form to four separate 10 mL volumetric flasks to obtain two sample solutions of each dosage form (equivalent to 100:4:2 µg/mL and 2000:80:40 µg/mL for GUF, BR and SAL, respectively and the volume was then adjusted using methanol.

For RP-HPLC method, samples solutions containing amount of GUF, BR and SAL equivalent to 1000:40:20 µg/mL, respectively were prepared by accurately transferring 1mL of each dosage form into two separate 10 mL volumetric flasks then the volume was completed to the mark with methanol. Further dilutions were performed in methanol to obtain two diluted solutions, the first contained concentrations equivalent to **25**: 1: 0.5 µg/mL for GUF, BR and SAL respectively and the other contained the studied drugs in the concentrations of 500: **20: 10** µg/mL, consequently.

#### Linearity and calibration curves

For TLC- densitometeric method, calibration curves were developed following the preparation of serial dilutions each of GUF, BR, SAL and GUL in the ranges of 50–800 µg/mL, 25–400 µg/mL, 25–400 µg/mL and 10–160 µg/mL, in order from their respective stock and working solutions (1000, 500, 500, 200 µg/mL for GUF, BR, SAL and GUL, in order) into four separate sets of 10 mL volumetric flasks. 10 µL of each sample was spotted in triplicates to the TLC plates to obtain concentrations equivalent to 0.50–8.00, 0.25–4.00, 0.25–4.00 and 0.10–1.60 µg/band for GUF, BR, SAL and GUL, consecutively, and then chromatographic separation was carried out as was explained before.

For RP-HPLC method, different samples in the ranges of 2–50 µg/mL for GUF, BR, SAL and GUL, respectively were prepared in methanol into four separate sets of 10 mL volumetric flasks using their previously prepared working solutions (100µg/mL) and then 30 µL of each prepared sample was injected to HPLC system. Separation was done following the chromatographic conditions illustrated before.

Peak areas for both methods were recorded for each drug for data analysis. Following that, calibration curves were constructed relating the determined peak area to the corresponding concentration, and regression equations were calculated.

#### Application to laboratory prepared mixtures

For TLC-densitometeric method, 10 µL of each prepared sample was applied to TLC plates in triplicates. While for HPLC method, 30 µL of the diluted samples was applied to HPLC system three times each. The chromatographic instructions of each method were then followed and regression equations were used to calculate concentrations of the studied components in the prepared mixtures.

#### Application to pharmaceutical formulation

For TLC-densitometeric method, 10 µL of each dosage form prepared samples was applied six times on TLC plates, while for HPLC 30 µL of each sample was injected to HPLC system six times each.

The developed methods were then applied following the conditions illustrated under TLC-densitometeric and HPLC chromatographic conditions. Peak areas were recorded for each drug for both methods, and the corresponding concentrations in the prepared pharmaceutical dosage form solutions were calculated using the computed regression equations. Additionally, for each drug, the standard addition procedure was carried out on three distinct levels using the two available dosage forms to test the accuracy of the methods.

## Results and discussion

Pharmaceutical industry has evolved over the years; its goal was to develop naturally or synthetically extracted drugs that improve public health. One aspect of concern has remained constant: the purity of the drug, which is vital to both its quality and safety. As a result, it was necessary to ensure the purity of the drugs and the absence of any harmful impurities. The analysis of pharmaceuticals and their related impurities have become a topic of interest [28].

This research included In-Silico study of toxicity profile and pharmacokinetic properties of GUL. Moreover, the work aimed to analyze two pharmaceutical dosage forms; Ventocough ® syrup (with and without sugar) which combines three active ingredients: GUF, BR and SAL in addition to propyl paraben and sodium benzoate as inactive ingredients by TLC-densitometeric and HPLC methods. Additionally, the work aimed to enhance the sensitivity of the methods to be able to quantify GUF impurity with the highest possible sensitivity.

### Pharmacokinetic and toxicological properties of guaiacol using ADMET Predictions

Predicting the toxicological features for GUL can aid in determining the significance of analysis of the studied drugs in its presence. The pkCSM [29] software was utilized in this study to predict the toxicological properties of GUL such as mutagenicity and hepatotoxicity. Additionally, the human pharmacokinetic data of GUL like absorption, distribution, metabolism, and excretion were assessed for the first time.

It was resulted that, GUL showed significant intestinal absorption value indicating high bioavailability to bloodstream which may result in danger effect on human health. Additionally, GUL can be considered as CYP1A2 inhibitor so it may be involved in drug-drug interactions as well as oxidative stress. Additionally, it had high skin permeability and Caco-2 cell line permeability (expect the oral absorption).

For toxicity profile, GUL can be considered as skin sensitizer and it showed T.Pyriformis toxicity. These findings ensured GUL toxicity, all gathered data are displayed in Table 2.

**Table 2:** Results of ADMET toxicity profile of guaiacol

Parameters		Guaiacol	Numerical Unit	Reference range
Absorption	Water solubility	− 1.11	Numeric (log mol/L)	Solubility increased by decreasing log S
	Caco2 permeability	**1.28**	Numeric (log Papp in 10^–6^ cm/s)	High permeability > 0.90
	Intestinal absorption (human)	94.995	Numeric (% Absorbed)	High absorbed > 30% Poorly absorbed < 30%
	Skin Permeability	− 2.408	Numeric (log Kp)	Logkp > -2.5
	P-glycoprotein substrate	No	Categorical (Yes/No)	–
	P-glycoprotein I inhibitor	No	Categorical (Yes/No)	–
	P-glycoprotein II inhibitor	No	Categorical (Yes/No)	–
Distribution	VDss (human)	0.004	Numeric (log L/kg)	Log VDss is considered Low < -0.15 Log VDss is considered high > 0.45
	Fraction unbound (human)	0.47	Numeric (Fu)	High > 0.45
	BBB permeability	0.037	Numeric (log BB)	- Log BB < -1 poorly distributed to the brain - Log BB > 0.3 cross the BBB
	CNS permeability	− 2.091	Numeric (log PS)	- Log PS < -3 unable to penetrate CNS - Log PS > -2 penetrate CNS
Metabolism	CYP2D6 substrate	No	Categorical (Yes/No)	This can be positively correlated to the lipophilicity of the compound to metabolism related toxicity
	CYP3A4 substrate	No	Categorical (Yes/No)	
	CYP1A2 inhibitior	**Yes**	Categorical (Yes/No)	
	CYP2C19 inhibitior	No	Categorical (Yes/No)	
	CYP2C9 inhibitior	No	Categorical (Yes/No)	
	CYP2D6 inhibitior	No	Categorical (Yes/No)	
	CYP3A4 inhibitior	No	Categorical (Yes/No)	
Excretion	Total Clearance	0.243	Numeric (log ml/min/kg)	
	Renal OCT2 substrate	No	Categorical (Yes/No)	
Toxicity	AMES toxicity	No	Categorical (Yes/No)	
	Max. tolerated dose (human)	1.332	Numeric (log mg/kg/day)	Low ≤ 0.477 High > 0.477
	hERG I inhibitor	No	Categorical (Yes/No)	
	hERG II inhibitor	No	Categorical (Yes/No)	
	Oral Rat Acute Toxicity (LD50)	**1.877**	Numeric (mol/kg)	
	Oral Rat Chronic Toxicity (LOAEL)	**2.636**	Numeric (log mg/kg_bw/day)	
	Hepatotoxicity	No	Categorical (Yes/No)	
	Skin Sensitisation	**Yes**	Categorical (Yes/No)	
	T.Pyriformis toxicity	− 0.229	Numeric (log ug/L)	Not toxic < —0.5 Toxic > —0.5
	Minnow toxicity	1.561	Numeric (log mM)	Highly acute toxic < —0.3 Not highly acute toxic > —0.3

### Method optimization

#### Optimization of TLC-densitometeric method

**Fig. 1 a:**
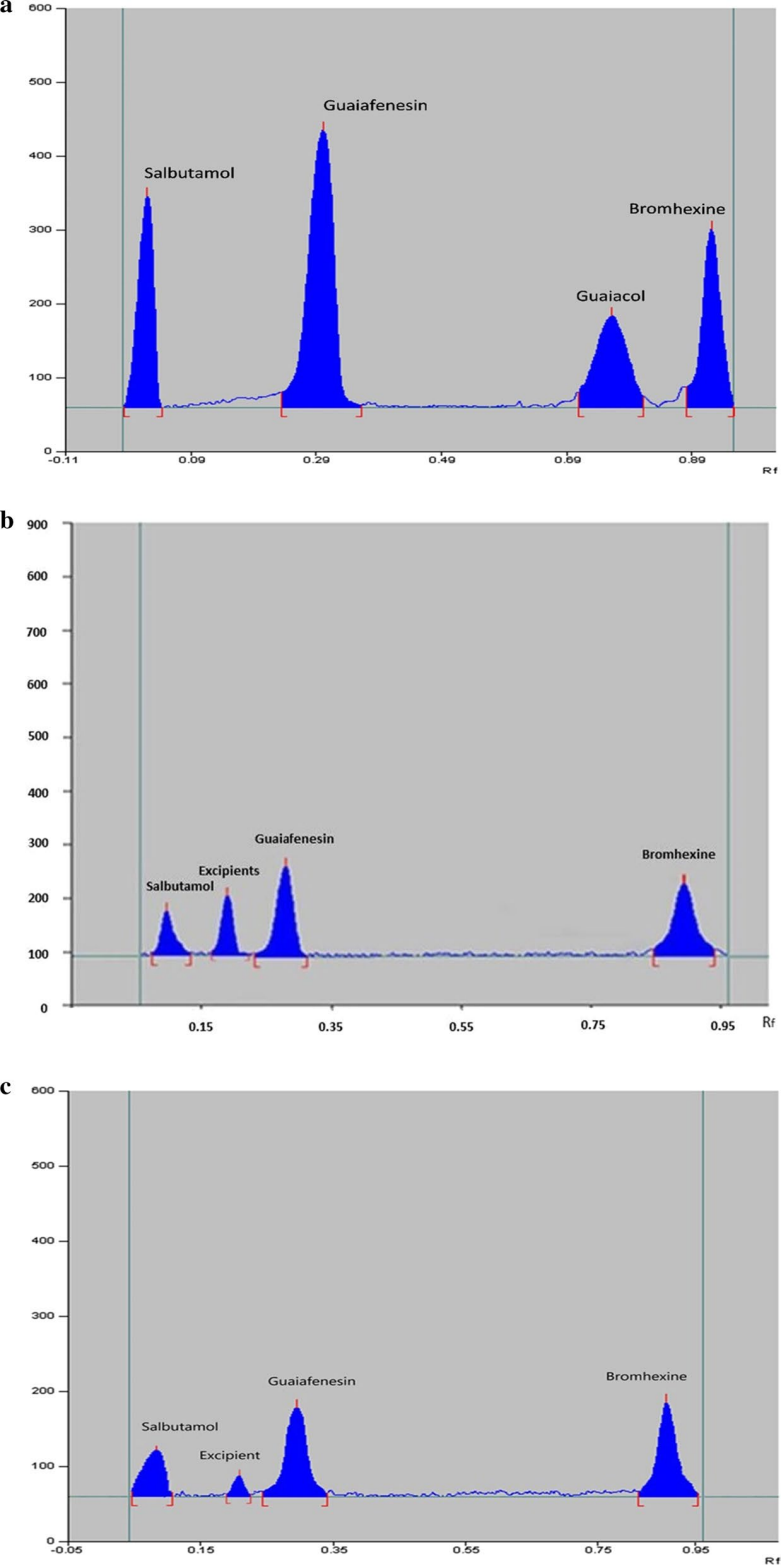
2D-TLC densitogram of a standard mixture of Salbutamol, Guaiafenesin, Guaiacol and Bromhexine, (Rf SAL: 0.07, Rf GUF: 0.29, Rf GUL: 0.76, and Rf BR: 0.92). **b** 2D-TLC densitogram of ventocough® syrup (with sugar) containing Salbutamol, Guaiafenesin, Bromhexine, and excipients, (Rf SAL: 0.07, Rf Excipients: 0.21, Rf GUL: 0.76, and Rf BR: 0.92). **c** 2D-TLC densitogram of ventocough® syrup (without sugar) containing Salbutamol, Guaiafenesin, Bromhexine, and Excipients, (Rf SAL: 0.07, Rf Excipients: 0.21, Rf GUL: 0.76, and Rf BR: 0.92)

TLC silica gel 60 F254 plates were used for the chromatographic separation. Different developing systems were tested to obtain the required resolution among the studied components, including (methylene chloride: ethyl acetate), (methylene chloride: hexane), (ethyl acetate: methanol), and (chloroform: methanol), all in the ratio of (5: 5, v/v). Methylene chloride: hexane system seemed to be promising; therefore optimization was continued using this mobile phase system. This solvent mixture showed good efficiency in the separation between dosage form excipient(s), GUF, GUL and BR and also in preventing GUL elution near the solvent front when it was used in the ratio of (6:5, v/v) (methylene chloride: hexane). Unfortunately, this developing system could not elute SAL from baseline. In order to improve R_f_ of SAL, polar solvents like methanol were added to the chosen developing system in different ratios, but no significant effect was observed. The next trial was to change pH of the system by using different volumes of glacial acetic acid and triethylamine separately. It was observed that SAL was eluted from the point of application with an acceptable R_f_ value on using triethylamine in the ratio of 0.3 mL without affecting the separation between other components. Finally, the optimum developing system was (methylene chloride: hexane: triethylamine) (6.0: 5.0: 0.3, by volume). Additionally, different saturation times were tested (5, 10, 15 and 30 min) and 10 min were found to be the most suitable time for saturation of chromatographic tank with the mobile phase mixture. In terms of detection wavelength, first trials started with scanning at several wavelengths, including 254, 275 and 310 nm. Optimum sensitivity with acceptable untailed peaks was obtained when scanning at 275 nm for SAL, GUF and GUL and 310 nm for BR. 2D densitograms showing complete separation among the studied components and the excipients with R_f_ values of (R_f_ SAL: 0.07, R_f_ Excipients: 0.21, R_f_ GUF: 0.29, R_f_ GUL: 0.76, and R_f_ BR: 0.92) are given in Fig. 1**.**

#### Optimization of HPLC method

**Fig. 2:**
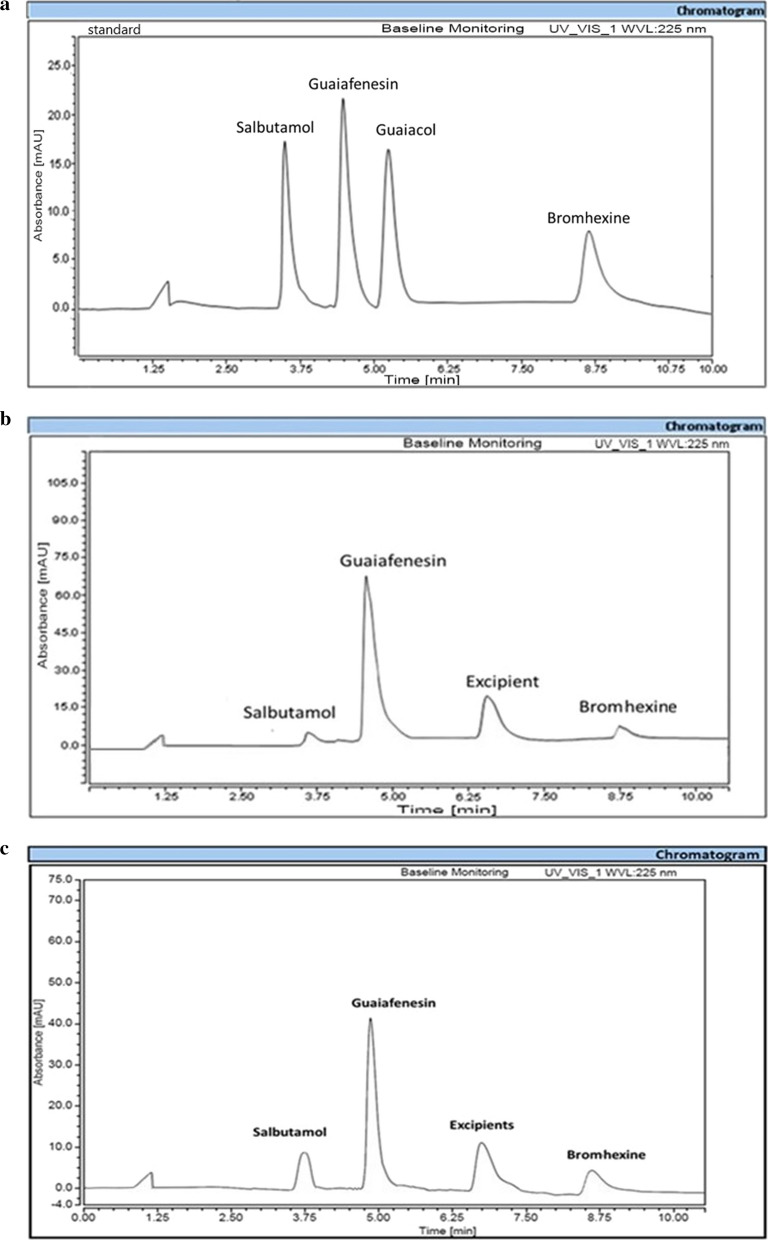
**a** HPLC method chromatogram of a standard mixture of Salbutamol, Guaiafenesin, Guaiacol and Bromhexine, (Rt SAL: 3.5, Rt GUF: 4.5, Rt GUL: 5.45, and Rt BR: 8.75 minutes)HPLC method chromatogram of a standard mixture of Salbutamol, Guaiafenesin, Guaiacol and Bromhexine, (Rt SAL: 3.5, Rt GU. F: 4.5, Rt GUL: 5.45, and Rt BR: 8.75 minutes). **b** HPLC chromatogram of the ventocough® syrup (with sugar), (Rt SAL: 3.5, Rt GUF: 4.5, Rt Excipient: 6.35, and Rt BR: 8.75 minutes). **c** HPLC chromatogram of the ventocough® syrup (without sugar), (Rt SAL: 3.5, Rt GUF: 4.5, Rt Excipient: 6.35, and Rt BR: 8.75 minutes)

Several columns with different polarities and dimensions were examined like X-Bridge® C_8_ column (250 × 4.6 mm, 5 μm), AOQUITY UPLC® BEH C_8_ (150 × 2.1 mm, 1.7 μm) and X-Bridge® C_18_ column (250 × 4.6 mm, 5 μm), the later was chosen. X-Bridge® C_8_ column (250 × 4.6 mm, 5 μm) and AOQUITY UPLC® BEH C_8_ (150 × 2.1 mm, 1.7 μm) were not used as they showed high retention time for BR at 20 and 25 min, respectively which was not accepted concerning the goal of this work. X-Bridge® C_18_ column (250 × 4.6 mm, 5 μm) showed good separation for all drugs with acceptable analysis time. Reviewing the literature, most of reported methods were depended on using methanol as an organic modifier while using 0.05 M disodium hydrogen phosphate solution as an aqueous solvent. Hence, trials began with a solvent mixture of methanol: 0.05M disodium hydrogen phosphate solutions in different ratios (80:20, v/v to 20: 80, v/v) using mobile phase flow rate of 1 mL/min. It was observed that increasing methanol more than 60% resulted in unresolved peaks while increasing the ratio of the used phosphate solution above 40% elongated the run time to more than 30 min. So this solvent mixture was used in the ratio of (60:40, v/v) that resulted in acceptable separation between SAL and GUF but bad separation between GUF and GUL. In order to completely separate GUF and GUL, it was reported that addition of triethylamine was essential [23]. When testing the effect of triethylamine ratio (0.1%-0.4%, v/v), it was found that 0.3% triethylamine when added to the organic modifier was sufficient to obtain complete resolution between GUF and GUL. It was worth mentioning that, pH of the used aqueous solvent significantly affected only the retention time as well as the peak shapes of SAL where pH 3 was the optimum value. In all the performed trials, the analysis time exceeded 15 min. In order to shorten the analysis time and improve BR retention time without affecting the separation efficiency, different flow rates were tested where no satisfied results were observed. Finally, flow rate programming was used by changing the flow rate during the analysis using an optimized program; details are given in Table 1. Complete separation with acceptable R_t_ were observed (R_t_ SAL: 3.5, R_t_ GUF: 4.5, R_t_ GUL: 5.45, and R_t_ BR: 8.75 min) and shown in Fig. 2.

### Method validation

The developed methods were validated according to ICH guidelines [30]. Different parameters were investigated including specificity, linearity, accuracy, robustness, system suitability, LOD and LOQ to ensure validity of the suggested methods for application in routine quality control work.

#### Linearity and calibration curves

Under optimum chromatographic conditions, linear calibration curves were constructed using eight different standard samples for each of the studied drugs. For TLC-densitometeric method, linearity was achieved for the proposed drugs in the ranges of 0.50–8.00 µg/band, 0.25–4.00 µg/band, 0.25–4.00 µg/band and 0.10–1.60 µg/band for GUF, BR, SAL and GUL where good correlation coefficients > 0.999 were obtained. While for HPLC method, nine different standard samples were used to establish linear calibration curves for each of the proposed drugs where linearity was established in the range of 2–50 µg/mL for all components with resulted correlation coefficients of 0.9999.

Linear regression parameters are presented in details in Table 3 while linearity curves are given in Additional file 1: Figs. S1 and S2. All of the regression equation parameters obtained confirmed the methods' linearity within the tested ranges.

**Table 3:** Regression and validation parameters of the proposed methods for determination of guaiafenesin, bromhexine, salbutamol and guaiacol

Parameters	TLC				HPLC			
	GUF	BR	SAL	GUL	GUF	BR	SAL	GUL
Calibration range	0.50–8.00 μg/band	0.25–4.00 μg/band	0.25–4.00 μg/band	0.10–1.60 μg/band	2.00–50.00 μg/mL			
Slope	1615.60	2779.20	1556.40	3723.30	0.2082	0.1328	0.1428	0.2031
Intercept	2547.20	2426.00	716.89	733.65	− 0.0560	− 0.1091	0.0907	− 0.3102
Correlation coefficient (r^2^)	0.9998	0.9998	0.9999	0.9999	0.9999	0.9999	0.9999	0.9999
Accuracy (%R)	98.59	99.84	99.76	99.82	99.77	99.83	99.09	99.20
Repeatability (%RSD)^a^	0.79	0.71	0.93	0.39	1.42	0.87	1.27	1.47
Intermediate precision(%RSD)^a^	1.00	0.81	1.32	0.99	1.77	1.04	1.34	1.54
LOD^b^	0.16	0.07	0.08	0.02	0.56	0.61	0.51	0.59
LOQ^c^	0.48	0.21	0.24	0.06	1.70	1.83	1.53	1.78

Where SD is the standard deviation of the intercept and slope is the slope of the calibration graph

^a^The intra-day and inter-day precision (n = 9), %RSD of three distinct concentrations; 1, 4 and 6 μg/band (for GUF), 0.5, 2, and 3 μg/band (for BR and SAL), 0.2, 0.8, and 1.4 μg/band (for GUL) for TLC-densitometeric method. For HPLC method, concentrations applied were; 3, 15 and 30 μg/ mL for all components

^b^LOD = (SD of the intercept /slope) × 3.3

^c^LOQ = (SD of the intercept /slope) × 10

#### Specificity

The specificity of the methods was examined by visual examination of chromatograms obtained from blank samples Additional file 1: Figs. S3 and S4, pure samples and those resulted from the tested dosage forms Figs. 1, 2. The chromatograms showed no interference between the separated drugs and the excipients. Additionally, complete separation was obtained between the main analytes and the studied impurity, confirming the stability indicating properties of the developed approaches. Moreover, results obtained when applying these approaches to the laboratory prepared mixtures, Table 4, were close to the correct values were assuring no evidence of interference between the separated components.

**Table 4:** Findings of the developed methods for the analysis of laboratory prepared mixtures, the two pharmaceutical dosage forms and the application of standard addition technique

	TLC	HPLC
Laboratory prepared mixtures^a^ (Mean ± %RSD)		
GUF BR SAL GUL	99.64 ± 1.27 99.51 ± 1.11 100.20 ± 1.06 99.90 ± 0.89	99.17 ± 2.31 98.16 ± 1.23 98.29 ± 1.59 100.3876 ± 1.34
Pharmaceutical formulation^b^ (With sugar) (Mean ± %RSD)		
GUF BR SAL	99.61 ± 1.61 94.16 ± 2.19 99.89 ± 1.52	101.44 ± 2.29 94.59 ± 2.46 99.34 ± 1.96
Pharmaceutical formulation^b^ (Without sugar) (Mean ± %RSD)		
GUF BR SAL	96.20 ± 2.00 97.52 ± 2.83 95.89 ± 1.63	96.72 ± 1.84 98.56 ± 2.16 96.95 ± 1.67
Standard addition^c^ (with sugar) (Mean ± STD)		
GUF BR SAL	99.21 ± 1.46 99.05 ± 1.13 99.04 ± 1.33	99.47 ± 1.19 99.28 ± 1.76 100.37 ± 1.37
Standard addition^c^ (without sugar) (Mean ± %RSD)		
GUF BR SAL	99.28 ± 1.38 98.69 ± 1.36 99.58 ± 1.49	99.29 ± 1.98 98.73 ± 1.57 97.61 ± 1.04

^a^Average of determinations of six laboratory prepared mixtures each was analyzed in triplicates

^b^Average of 6 determinations (For TLC-densitometeric method, the measured concentrations were 1.00, 0.80 and 0.40 for GUF, BR, and SAL, respectively. While for HPLC, Concentrations were 25, 20, 10 for GUF, BR and SAL, respectively)

^c^Average of 3 determinations of standard addition samples

For TLC, the added concentrations to the prepared dosage forms were: For GUF: 1.00, 2.00 and 3.00 µg /band, For each of BR or SAL: 0.2, 0.50 and 1.00 µg /band

For HPLC, the added concentrations were: For GUF:15, 20, and 25 µg /mL, For each of BR or SAL: 10, 15, and 20 for BR and SAL, in order)

#### Accuracy

Accuracy of the proposed methods was checked by determining three different standard concentrations in triplicates for each of the studied components. Mean percentage recoveries were employed to report accuracy of the methods, Table 3. The obtained recoveries ranged from 98.59 to 99.76% (for TLC densitometric method) and from 98.73 to 99.76% (for HPLC method). All these results confirmed good accuracy of the methods.

#### Precision

Precision of the developed methods was tested using three different concentrations of each component. Intraday precision (repeatability) involved analyzing each concentration three times on the same day, while interday precision (intermediate precision) involved analyzing each component three times on three separate days. The results were expressed as a percentage relative standard deviation (%RSD). All findings were < 2% confirming that the developed methods had good precision either on intraday or interday precision levels, Table 3.

#### Limit of detection and limit of quantitation

Sensitivity of the methods was evaluated by the determination of LOD and LOQ. They were calculated using the standard deviations (σ) of the intercept for the regression line and the slope (s) of the calibration curves, they were estimated as LOD: (3.3 × σ /s) and LOQ = (10 × σ /s) [31]. For TLC-densitometeric method, LOD calculation was resulted in 0.16, 0.07, 0.08 and 0.02 µg/band, while for LOQ, it was resulted in 0.48, 0.21, 0.24 and 0.06 µg/band for GUF, BR, SAL and GUL, respectively. For the HPLC approach, LOD was determined to be 0.56, 0.61, 0.51, and 0.59 µg/mL, while LOQ was calculated to be 1.70, 1.83, 1.53, and 1.78 µg/mL, respectively, for GUF, BR, SAL, and GUL Table 3. All the obtained findings ensured the good sensitivity of the methods.

#### Robustness

The method was ensured to be robust by checking the effect of small chromatographic changes in method parameters on the performance of the method. Two conditions were changed intentionally in small portions; triethylamine volume and the saturation time for TLC-densitometeric method. For triethylamine, three different volumes were tested (0.2, 0.3 and 0.4 mL) while for saturation time of the mobile phase, it was examined over three different intervals (5, 10 and 15 min). %RSD values were calculated for the resulted R_f_ values. Results in Table 5 showed no significant changes on R_f_ values on performing the tested changes. Concerning to RP-HPLC method, influence of % methanol in the mobile phase (± 2%, v/v) and wavelength (± 2 nm) were examined. Results in Table 5 revealed that the small intended changes under test did not affect the R_t_ of the separated analytes significantly. All results in Table 5 confirmed robustness of the methods.

**Table 5:** Results of robustness of the developed methods

Analyte		TLC-densitometric method		HPLC method	
		Triethylamine ratio ± 0.1mL	Mobile phase saturation time ± 5 min	Methanol ratio ± 2%	Wavelength ± 2 nm
GUF	%RSD^a^	0.11	0.11	0.09	0.05
BR		0.01	0.01	0.03	0.05
SAL		0.65	0.43	0.07	0.03
GUL		0.04	0.01	0.07	0.04

a%RSD of the change in Rf value (for TLC-densitometric method) and the change in tR value (for HPLC method) values on changing some conditions

#### System suitability

The purpose of testing system suitability parameters is to assess the performance of the overall system. The performance of the system was verified by calculation of resolution (R_s_), selectivity (A), capacity factor (K ‘) and tailing factor (t) using standard chromatograms following the reported instructions [7, 32]. The results demonstrated the system’s ability to separate the studied drugs from the various interfering substances and impurities. All results were within acceptable limits Table 6**.**

**Table 6:** System suitability parameters for the developed methods

Parameters	TLC	Reference range [32]	HPLC	Reference range [7]
Resolution (R_S_)	R_s__(SAL-EXP)=_ 1.53 R_s__(EXP-GUF)=_ 1.54 R_s__(__GUF-GUL)=_ 4.27 R_s__(__GUF-BR)=_ 1.88	≥ 1.5	R_s (SAL-GUF)=_ 1.6 R_s__(GUF-GUL)=_ 1.53 R_s__(__GUL-EXP)=_ 2.14 R_s__(__EXP-BR)=_ 3.8	≥ 1.5
Selectivity (α)	α _(SAL-EXP)=_ 2.42 α _(EXP-GUF)=_ 1.58 α _(__GUF-GUL)=_ 2.96 α _(__GUF-BR)__=_ 1.20	≥ 1	α _(SAL-EXP)=_ 1.52 α _(EXP-GUF)=_ 1.28 α _(__GUF-GUL)=_ 1.33 α _(__GUF-BR)__=_ 1.84	≥ 1
Capacity Factor (K’)	K’ _(SAL)=_ 19.00 K’ _(GUF)=_ 2.44 K’ _(__GUL_)_=_ 0.33 K’ _(__BR_)_=_ 0.10	> 0.1	K’ _(SAL)__=_ 1.80 K’ _(GUF)__=_ 2.60 K’ _(__GUL_) _=_ 3.36 K’ _(__BR_)_=_ 6.00	1–10
Tailing Factor (T)	T _(SAL)=_1.00 T _(GUF)=_1.10 T_( GUL_)_=_1.05 T _( BR_)_=_1.02	> 1.5	T _(SAL)__=_ 1.20 T _(GUF)__=_ 1.13 T_(__GUL_) _=_ 1.05 T _(__BR_)_=_ 1.12	1–1.5
Number of theoretical plates (N)	–	–	N _(SAL)__=_1443 N _(GUF)__=_1817 N_(__GUL_) _=_2562 N _(__BR_)_=_2946	The higher the number, the more efficient separation
Height Equivalent to a Theoretical Plate (mm) (HETP)	–	–	N _(SAL)__=_0.17 N _(GUF) =_0.14 N_(__GUL_) _=_0.09 N _(__BR_)_=_0.08	The lower the value, the more efficient separation

### Results of analysis of pharmaceutical dosage forms

Application of the proposed methods for analysis of the studied analytes in ventocough ® syrup (with sugar and without sugar) was carried out in order to assess their validity and all the obtained recoveries agreed with the acceptable limits (90–110%), Table 4**.** The results ensured that syrup labeled excipients did not interfere with the analysis of the labeled active ingredients, confirming selectivity of the suggested methods. Additionally, accuracy of the developed methods was further assured by applying standard addition technique at three different levels and expressed as mean recovery, Table 4**.** The findings of application of this technique to the available dosage forms were all close to 100%, ensuring no interference from labeled additives and confirming methods accuracy.

### Statistical comparison with the reported methods

Statistical comparison was established between the proposed and the reported method [27], Table 7**.** The computed t-values and F-values were lower than those of the reported methods, indicating that there was no significant difference between the suggested and reported method.

**Table 7:** Statistical comparison between the developed method and the reported one using pure samples of the studied drugs

Parameters	GUF		BR		SAL		Reported method [27]		
							GUF	BR	SAL
	TLC	HPLC	TLC	HPLC	TLC	HPLC			
Mean	98.59	99.77	99.84	99.83	99.76	99.09	99.17	100.89	98.89
SD	1.49	1.24	0.84	1.27	1.22	1.41	2.29	1.43	1.42
Variance	2.22	1.55	0.72	1.63	1.50	1.98	5.26	1.93	2.02
N	9	9	9	9	9	9	8	8	8
Student's t-test (2.130)	0.63	0.67	2.01	1.72	1.35	0.29	–	–	–
F-test (3.500)	2.37	3.39	2.70	1.19	1.34	1.02	–	–	–

Moreover, comparison between the performance of the suggested approaches and reported one [27] was carried out, Table 8**.** The comparison revealed that, the suggested approaches are efficient, selective, and sensitive than the reported chemometric methods. Hence they can be used as successful alternatives to the high money consuming approaches such as LC/MS/MS method.

**Table 8:** Comparison between the developed methods and the reported one

Item for comparison	Methods under comparison		
	The developed method TLC-densitometric method	The developed method RP-HPLC method	Chemometric methods [27]
Linearity range	-GUF: 0.5–8.0 µg/band -BR: 0.25–4.0 µg/band -SAL: 0.25–4.0 µg/band -GUL: 0.1–1.6 μg/band	-GUF: 2–50 μg/mL -BR: 2–50 μg/mL -SAL: 2–50 μg/mL -GUL: 2–50 μg/mL	-GUF: 10.0–30.0 μg/mL -BR: 10.0–30.0 μg/mL -SAL: 10.0–30.0 μg/mL -GUL:6.0–10.0 μg/mL
Solvents used	hexane: methylene chloride: triethylamine (5:6:0.3, by volume)	0.05M disodium hydrogen phosphate pH 3 with aqueous phosphoric acid: methanol (containing 0.3% triethylamine) (40:60, v/v)	Methanol
Comments	-It is the first TLC-densitometeric method for the determination of the studied drugs and impurity without interference from excipients -It has low energy and money consumption -It has low waste production -It has short analysis time	-It is the first developed stability indicating HPLC method for quantitation of the impurity - The run time is less than 10 min	- It has the lowest energy and money consumption - The sensitivity is lower than the developed methods according to the linearity ranges - It measures the drugs in the presence of the excipients without separation of the excipients, so it always needs certain modifications on the method to remove the interference from excipients
	-They have higher sensitivity for the studied drugs as well as the impurity -Insilico study for the selected impurity to study the toxicity profile and the pharmacokinetic properties -Complete separation of drug from the excipients with minimum sample pretreatment steps		

## Conclusion

Currently, developing sensitive analytical methods for quantitation of active pharmaceutical ingredients and their reported impurities becomes an issue of concern in pharmaceutical industries. Therefore, this work aimed to develop and optimize two novel chromatographic methods for simultaneous determination of BR, SAL, GUF, and GUL (GUF impurity). Methods validation was done according to ICH guidelines and all results were within the acceptable limits. In addition, the proposed methods were proved to be efficient and accurate for estimating the combined active ingredients in Ventocough syrup® even in presence of syrup excipients. TLC densitometric method can be selected when money and time are important factors especially in developing countries while RP-HPLC one can be used as an efficient alternative to the money consuming LC/MS approach. Furthermore, the work was extended to predict the pharmacokinetic behavior and toxicity profile of GUL.

### Supplementary Information

**Additional file 1: Fig. S1.** Calibration curve for TLC-densitometric method relating integrated peak area x 10 ^-3^ of GUF (**A**), BR (**B**), SAL (**C**) and GUL (**D**) with the corresponding concentrations in the range of 0.5-8.0, 0.25-4.0, 0.25-4.0, and 0.1-1.6 µg/band, respectively. **Fig. S2.** Calibration curve for RP-HPLC method relating integrated peak area x 10 ^-3^ of GUF (A), BR (**B**), SAL (C) and GUL (D) with the corresponding concentrations in the range of 2-50 µg/mL for all the proposed drugs. **Fig. S3.** 2D-TLC densitogram of blank sample. **Fig. S4.** HPLC chromatogram of blank sample.

## Data Availability

The data that support the findings of this study are available from the corresponding author (Nada Sayed Abdelwahab) upon reasonable request.

## Abbreviations

BR: Bromhexine
GUF: Guaiafenesin
SAL: Salbutamol
GUL: Guaiacol
TLC: Thin layer chromatography
HPLC: High performance liquid chromatography
ICH: International Council for Harmonization of Technical Requirements for Pharmaceuticals for Human
DGP: Delta Grand Pharma
