# Supplementary material for: Chromatographic analysis of triple cough therapy; bromhexine, guaiafenesin and salbutamol and pharmaceutical impurity: in-silico toxicity profile of drug impurity
Source: BMC Chem. 2024 Jan 27;18(1):19. doi: 10.1186/s13065-024-01122-5 (PMC10821540; doi:10.1186/s13065-024-01122-5)
Supplement: Supplementary file 1 — Additional file 1: Fig. S1. Calibration curve for TLC-densitometric method relating integrated peak area x 10 -3 of GUF (A), BR (B), SAL (C) and GUL (D) with the corresponding concentrations in the range of 0.5-8.0, 0.25-4.0, 0.25-4.0, and 0.1-1.6 µg/band, respectively. Fig. S2. Calibration curve for RP-HPLC method relating integrated peak area x 10 -3 of GUF (A), BR (B), SAL (C) and GUL (D) with the corresponding concentrations in the range of 2-50 µg/mL for all the proposed drugs. Fig. S3. 2D-TLC densitogram of blank sample. Fig. S4. HPLC chromatogram of blank sample. [file 13065_2024_1122_MOESM1_ESM.docx]

**
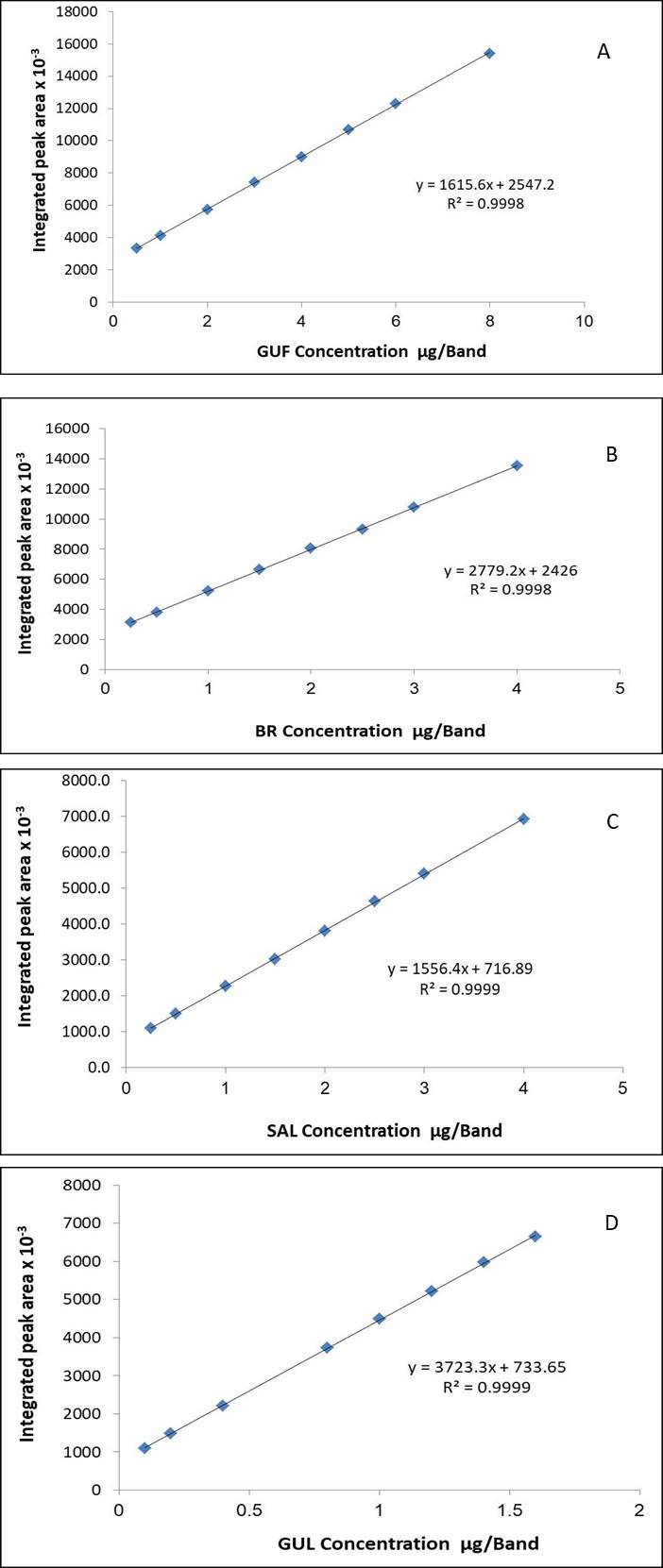
Additional file 1**

**Figures**

**Figure S1** Calibration curve for TLC-densitometric method relating integrated peak area x 10 ^-3^ of GUF (A), BR (B), SAL (C) and GUL (D) with the corresponding concentrations in the range of 0.5-8.0, 0.25-4.0, 0.25-4.0, and 0.1-1.6 µg/band, respectively.


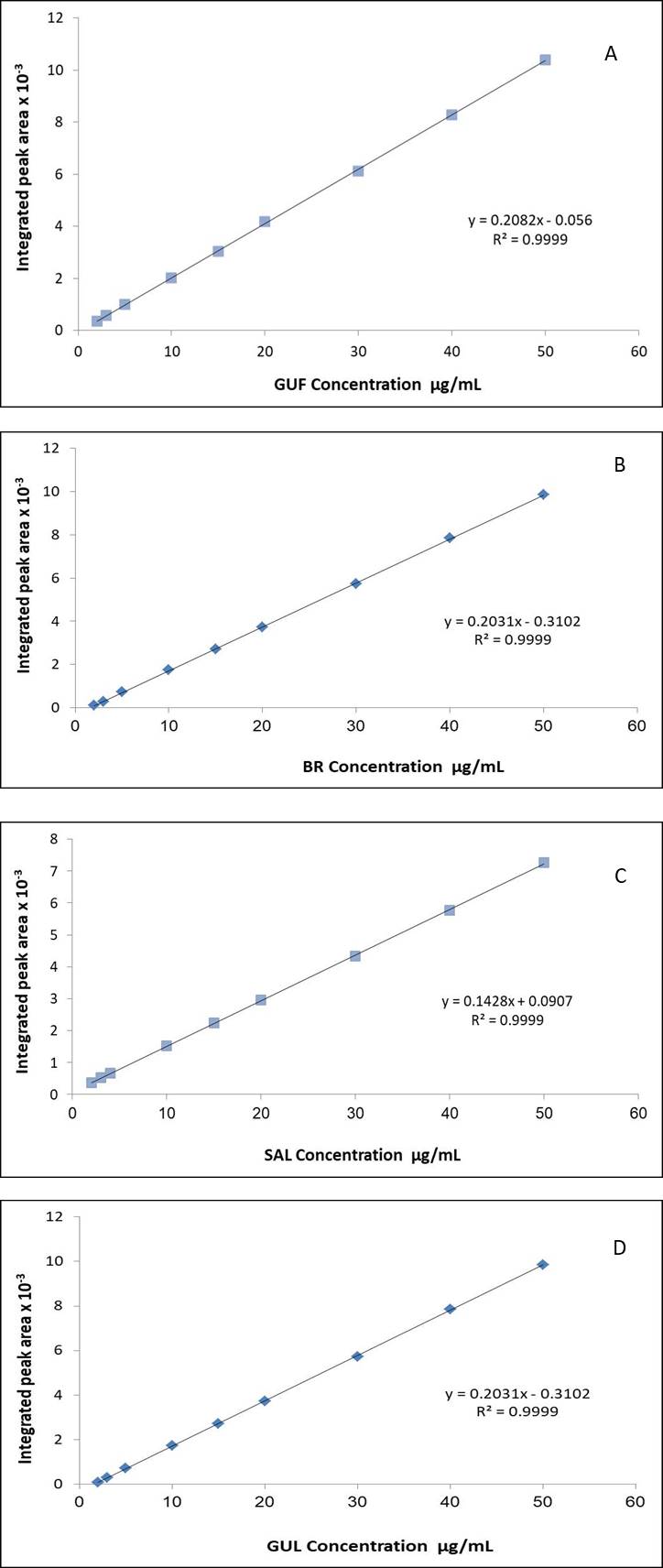


Figure S2 Calibration curve for RP-HPLC method relating integrated peak area x 10 ^-3^ of GUF (A), BR (B), SAL (C) and GUL (D) with the corresponding concentrations in the range of 2-50 µg/mL for all the proposed drugs.

**
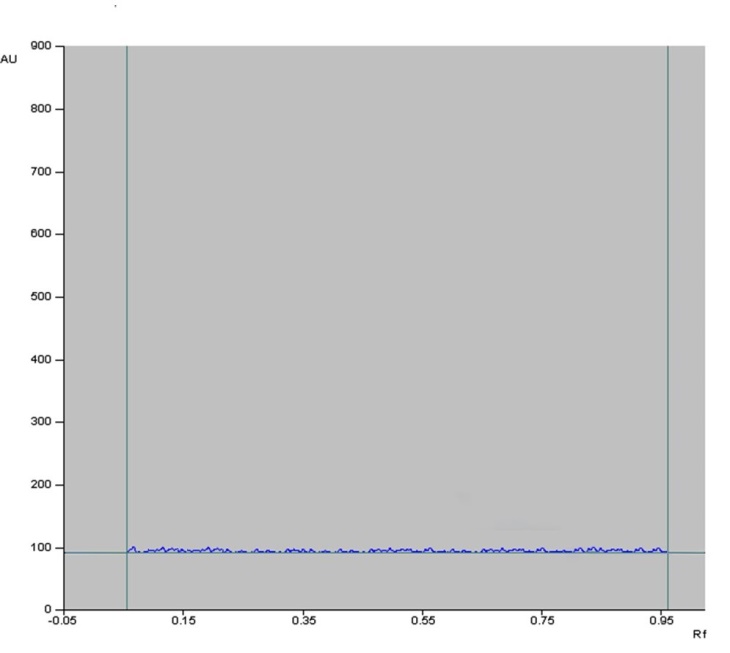
Figure S3** 2D-TLC densitogram of blank sample


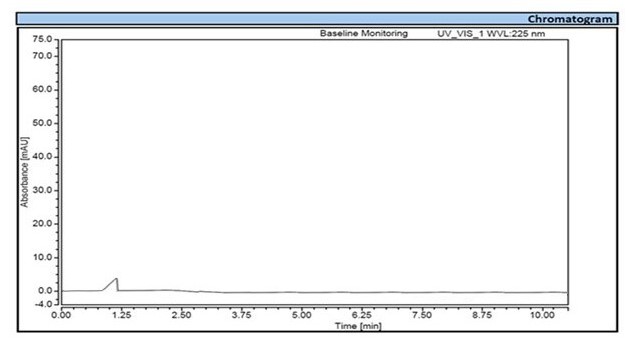


**Figure S4** HPLC chromatogram of blank sample
